# Extended score interval in the assessment of basic surgical skills

**DOI:** 10.3402/meo.v20.25819

**Published:** 2015-01-29

**Authors:** Stefan Acosta, Dan Sevonius, Anders Beckman, on behalf of the Swedish steering committee of the Basic Surgical Skills course, Swedish Surgical Society

**Affiliations:** 1 Vascular Centre, Skåne University Hospital, Malmö, Sweden; 2 Department of Surgery, Skåne University Hospital, Malmö-Lund, Sweden; 3 Department of General Medicine, Skåne University Hospital, Malmö, Sweden

**Keywords:** surgical education, basic surgical skills, residents, score interval, assessment

## Abstract

**Introduction:**

The Basic Surgical Skills course uses an assessment score interval of 0–3. An extended score interval, 1–6, was proposed by the Swedish steering committee of the course. The aim of this study was to analyze the trainee scores in the current 0–3 scored version compared to a proposed 1–6 scored version.

**Methods:**

Sixteen participants, seven females and nine males, were evaluated in the current and proposed assessment forms by instructors, observers, and learners themselves during the first and second day. In each assessment form, 17 tasks were assessed. The inter-rater reliability between the current and the proposed score sheets were evaluated with intraclass correlation (ICC) with 95% confidence intervals (CI).

**Results:**

The distribution of scores for ‘knot tying’ at the last time point and ‘bowel anastomosis side to side’ given by the instructors in the current assessment form showed that the highest score was given in 31 and 62%, respectively. No ceiling effects were found in the proposed assessment form. The overall ICC between the current and proposed score sheets after assessment by the instructors increased from 0.38 (95% CI 0.77–0.78) on Day 1 to 0.83 (95% CI 0.51–0.94) on Day 2.

**Discussion:**

A clear ceiling effect of scores was demonstrated in the current assessment form, questioning its validity. The proposed score sheet provides more accurate scores and seems to be a better feedback instrument for learning technical surgical skills in the Basic Surgical Skills course.

Surgical competence is dependent on technical skills as well as non-technical skills such as decision making, communication, team work, and leadership (1). Effective teaching and learning in technical surgical skills courses should follow the pedagogic principles of constructive alignment (2), where there is harmony among goal-directed teaching, teaching activities, and assessment. It is well known that assessment per se drives learning (3). The form of assessment, however, summative or formative, serves different purposes. It seems that elements of both summative and formative assessment may be beneficial in learning (4), whereas regular less stressful formative assessments may be better in retention of technical skills (5).

The Basic Surgical Skills course has adopted the teaching concept of one safe and standardized surgical technique for the most elementary skills. The course was introduced from Great Britain to Sweden in 2002. Since then, the course has been modified to suit the conditions in Sweden, and the course is mainly intended for surgical trainees in their first year of training. The aims and learning activities of the course have been modified, whereas the assessment score sheet has remained the same. The assessment score sheet (Appendix 1) has a very narrow score interval, scores 0–3, with little possibilities to reflect training progression and proper constructive feedback to the trainees. Furthermore, a score of ‘0’ has to our knowledge not been given during our courses, nor has anybody failed, indicating that assessment at this level of training should be formative rather than summative. Indeed, it was decided in a national steering meeting to revise the protocol into an entirely formative assessment score sheet and to compare the proposed wider score interval of 1–6 (Appendix 2), adopted from the direct observational procedural skills (DOPS) method (6, 7), against the current score sheet. The aim of this study was to analyze the trainees’ scores in the current and the proposed score sheets, assessed by the instructors, the external observers, and by the trainees themselves, and to estimate inter-rater reliability between the two score sheets.

## Methods

Sixteen participants were evaluated during the first and second day of the Basic Surgical Skills course (17–19 of April 2013) at Practicum, Lund, Skåne University Hospital, Sweden. All instructors and external observers recruited for this study were experienced instructors. The instructors and external observers were informed both in writing and orally about the revised proposed protocol 1 week before the start of the study and at the start of the study. The course participants were informed at the start of the study. One instructor and one observer were assigned to independently assess four participants in the four working stations according to protocol made up before the start of study. The instructors were instructor the first day and observer the second day; vice versa for the observers. In all, there were 11 instructors or observers, of whom 1 was female. Technical skills of each specific task were assessed using the current and the proposed evaluation form independently by the instructor, external observer, and the course participants themselves (self-assessment). All score sheets were collected by the principal investigator at the end of Day 1 and Day 2 to reassure independent scoring. Oral and written scores were given at the end of each forenoon or afternoon session by the respective instructors to each participant. In this way, formal assessments were performed four times during the study. All 96 evaluation forms were completed. In each evaluation form, 17 tasks were assessed. Oral feedback from the trainees to the teachers were given at the end of each day and written feedback according to the participant course evaluation form returned to the teachers immediately after the end of the course.

### Statistics

Age in women and men were defined in median age (range). Differences between groups were evaluated with the Mann–Whitney *U* test, and related samples with the Wilcoxon-signed rank test, and *p* <0.05 was considered significant. A floor or ceiling effect was considered to be present when more than 15% of participants received the lowest or the highest score, respectively (8). The inter-rater reliability (i.e., the consistency in the rating of subjects, although each subject is not provided exactly the same rating by all assessors) between the current and the proposed score sheets was evaluated with ICC with 95% confidence intervals (CI) (9). An ICC value >0.7 was regarded as satisfactory (10). In each score sheet, eight scores were assigned on the first day and nine scores were assigned on the second day. The overall ICC in the current and the proposed score sheet for each trainee during Day 1 (8×16=128 correlations) and Day 2 (9×16=144 correlations), respectively, by the instructors, the observers, and the trainees themselves, was calculated, and the reliability analysis was performed after entering, for instance, the instructors’ scores in the current and the proposed score sheet of all trainees on Day 1. The mean score of the specific tasks, namely ‘instrument handling’, ‘knot tying’, and ‘suture technique’, were calculated for all four time points based on the current and the proposed assessment score sheet, respectively, when written evaluation took place, and development of acquired scores was graphically displayed. Analysis was performed in SPSS, version 20.0, and Excel.

## Results

### The course participants

There was no difference in the age between the nine male and seven female participants with a median age of 35 years (range 28–43) and 31 years (range 29–39), respectively (*p*=0.41). The median time of experience in a surgical department was 5 (range 0.5–34) months, without gender difference (*p*=0.92). The two most experienced trainees had 13 and 34 months of experience, respectively, whereas all other trainees had less than 9 months of experience.

### Inter-rater reliability between the current and the proposed score sheets

The ICC between the current and the proposed score sheet was lower on Day 1 than on Day 2, particularly for the instructors (Table 1). The ICC between the two score sheets after assessment of knot tying on the first day morning were 0.50 (−0.43–0.82), 0.50 (−0.43–0.82), and 0.80 (0.43–0.93) by instructors, observers, and trainees, respectively. The ICC between the two score sheets after assessment of knot tying on Day 2 afternoon were 0.92 (0.78–0.97), 0.70 (0.13–0.89), and 0.75 (0.28–0.91) by instructors, observers, and trainees, respectively.

**Table 1:** The overall inter-rater reliability of instructors, observers, and self-assessment of trainees when scoring in the current and proposed assessment sheet during Day 1 and Day 2.

Assessor	Day 1 ICC (95% CI) (*n*=128)	Day 2 ICC (95% CI) (*n*=144)
Instructors (*n*=16)	0.38 (−0.77–0.78)	0.83 (0.51–0.94)
Observers (*n*=16)	0.68 (0.08–0.89)	0.69 (0.10–0.89)
Self-assessment (*n*=16)	0.77 (0.33–0.92)	0.83 (0.52–0.94)

ICC=Intra-class correlation.

### Assessment of repeated technical skills

**Fig. 1:**
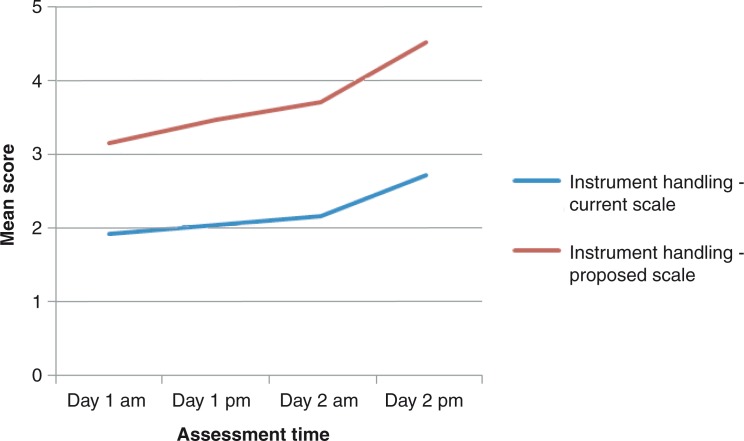
Instrument handling: assessment by instructors.

**Fig. 2:**
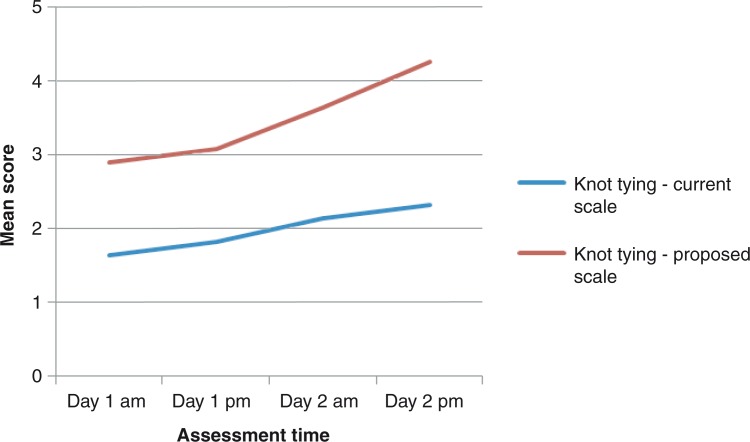
Knot tying: assessment by instructors.

**Fig. 3:**
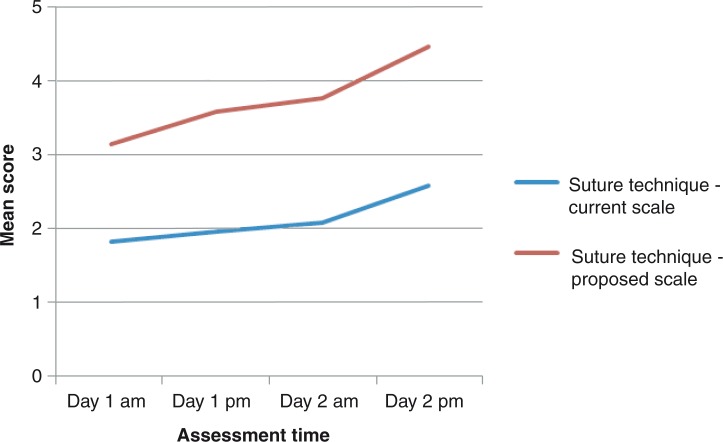
Suture technique: assessment by instructors.

**Fig. 4:**
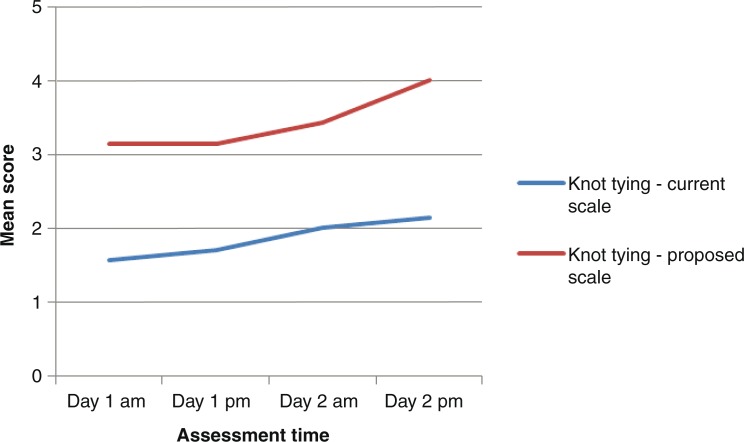
Knot tying: assessment by female trainees.

**Fig. 5:**
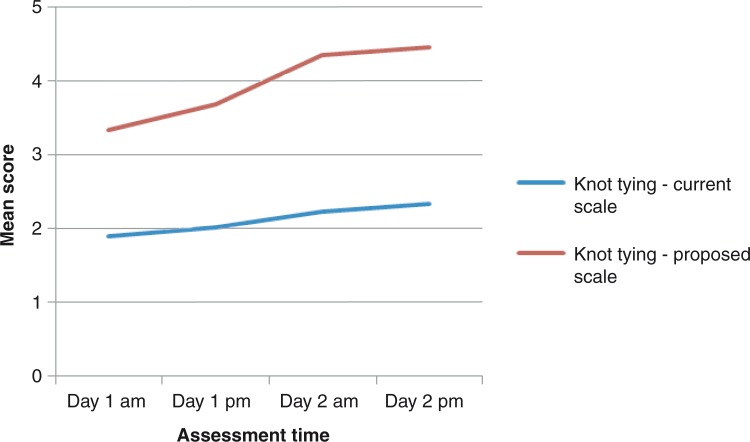
Knot tying: assessment by male trainees.

The progression lines toward higher scores in instrument handling, knot tying, and suture technique throughout the study was steeper for the proposed score sheet compared to the current score sheet (Figs. 1–5). The distribution of scores given by the instructors for assessment of ‘knot tying’ according to the current score sheet at the first and the last time point was score 1 (*n*=6) and score 2 (*n*=10) at the first time point compared to score 2 (*n*=11) and score 3 (*n*=5) at the last time point (*p*=0.001). The highest score, score 3, was given in 31% (5/16) at the last time point. The distribution of scores given by the instructors for assessment of ‘knot tying’ according to the proposed score sheet at the first time point was score 2 (*n*=3), score 3 (*n*=12), and score 4 (*n*=1), and at the second time point was score 4 (*n*=4) and score 5 (*n*=12), (*p*<0.001). The distribution of scores given by the instructors for assessment of ‘bowel anastomosis end to end’ on Day 2 morning and ‘bowel anastomosis side to side’ on Day 2 afternoon, according to the current score sheet, was score 2 (*n*=14), score 3 (*n*=2), and score 2 (*n*=6), score 3 (*n*=10), respectively (*p*=0.011). The highest score, score 3, was given in 62% (10/16) at ‘bowel anastomosis side to side’. The distribution of scores given by the instructors for assessment of ‘bowel anastomosis end to end’ on Day 2, morning, and ‘bowel anastomosis side to side’ on Day 2, afternoon, according to the proposed score sheet was score 3 (*n*=5), score 4 (*n*=10), score 5 (*n*=1), and score 3 (*n*=2), score 4 (*n*=5), score 5 (*n*=9), respectively (*p*=0.001).

### Gender perspectives on self-assessments

The female participants assessed themselves with lower scores than male participants (Fig. 4 and Fig. 5), but this was only significant for knot tying at time point 3 in the proposed score sheet: The median score for females and males after self-assessment in knot tying at time point 3 in the proposed score sheet was 3 (range 3–4) and 4 (range 4–5), respectively (*p*=0.016), whereas the instructors scored 4 (range 3–4) and 4 (range 3–4), respectively (*p*=0.61).

## Discussion

The proposed score sheet has a more extended score interval than the current score sheet, making it possible to better use the scores as a more dynamic feedback (11–13) instrument, with a larger room for improvement of scores in repeated assessments of the same task and retention of acquired skills. The proposed score sheet can be a more precise steering tool, better reflecting actual level of acquired skills. The inter-rater reliability between the instructors’ current and proposed score sheet were low on Day 1, probably due to a very limited room for different scores in the current score sheet. The scores in the current score sheet were therefore relatively higher than in the proposed score sheet on Day 1. Training improved scores relatively more in the proposed as opposed to the current assessment form. Hence, the higher intraclass correlation on Day 2 reflects a better agreement between the two score sheets.

The current score interval of 0–3 is too narrow, whereas a score of ‘0’ is a strong symbol for failure. It is an unnecessary repressive score in a formative assessment context where the open stimulating interaction between the teacher and the trainee is important to maintain (14). It is important to distinguish such a repressive score from negative feedback, which may be as effective on surgical performance and motivation as on positive feedback (15). ‘No instructor feedback at all’ is associated with inferior skills performance compared to when instructor feedback is given (16). The other extreme, the perfect score of ‘3’, should in practice be considered nearly impossible to achieve for trainees. Nevertheless, perfect scores were given to some extent at the final assessment in various technical learning activities in the current assessment sheet and, indeed, a clear ceiling effect was noted. If the participant's skills deteriorate during training, temporarily or permanently during the course, it may be difficult to lower a score from ‘2’ to ‘1’. Hence, the score interval of 1–2 in the current score sheet has to be replaced by a revised score sheet. In accordance with our opinion, the Royal College of Surgeons in Great Britain has found it necessary to revise the Intercollegiate Basic Surgical Skills assessment scale and feedback. The revised, slightly extended scale is, however, similar to the old scale, where the scale interval has been altered from 0–3 to 1–5. The interpretation of the revised scores of 1–2 and 4–5 corresponds to the old scores of 0–1 and 2–3, respectively, whereas the revised score ‘3’ has been added. This intermediate revised score of ‘3’ means that the participant performs satisfactorily, identifies occasional errors, and needs some supportive assistance to correct these errors. There are, for example, extended score intervals of 1–9 (1–3 unsatisfactory, 4–6 satisfactory, and 7–9 excellent) that may be even better, although not proven, in the teaching of manual technical skills (17). The downsides, however, of increasing the score intervals are the difficulties in developing defined criteria for each score and for each skill or procedure.

Assessment of technical surgical skills in trainees at the beginning of their specialization is usually based on procedure-specific checklists or global rating scales that may be applied for any type of surgical procedure (18). The most valid and used global rating scale to test operative technical skills is the Objective Structured Assessment of Technical Skill (OSATS), originally developed for bench model simulations (19). The global seven item five-point rating scale (1–5) assess the trainees’ respect for tissue, time and motion, instrument handling, instrument knowledge, use of assistant, flow of operation and knowledge of specific procedure. This global rating scale has been shown to be a more appropriate method to test technical skills than procedure-specific checklists (20). The global rating form in OSATS is, however, not directly applicable to the Basic Surgical Skills course, since only ‘instrument handling’ and ‘use of assistant’ has been defined in OSATS, whereas ‘knot tying’, ‘suture technique’, ‘bowel anastomosis’, ‘abdominal wall closure’, ‘arteriography’, and ‘patch anastomosis’ has not been defined. Development of defined assessment criteria for the scores 1–5 or 1–6 for each skill or procedure in the Basic Surgical Skills course may be helpful for instructors to give more accurate scores, improving inter-observer agreement, and to be able to provide more precise and understandable feedback to the trainees. The assessment form for trainees is often mixed, both formative and summative as for the global rating form in OSATS. Formative assessment may, however, be better for trainees, stimulating learning in a more relaxed manner together with the instructor, avoiding excessive stress associated with summative assessment (21). For this purpose, the revised proposed score sheet in the current study does not assess whether the trainee has ‘passed’ or ‘failed’.

The female trainees rated themselves lower than men in skill assessment, although non-significant in several learning activities, which may be due to a type-2 statistical error. This discrepancy in self-assessment between genders, however, is well known (22) and may be due to underestimation of the level of acquired skills by the females, overestimation by the males, or a combination of both. Scientific reports need to take this aspect into account. Consideration of gender differences in self-perception is also important when providing feedback to female surgical residents. The instructors should be particularly reassuring and convincing in their feedback to well-performing females with low self-esteem to be able to maximize their learning curve. Higher year of training, older age, and non-European nationality was reported to be even more predictive, than gender, of accuracy in self-prediction and self-assessment (23).

A larger sample size would have been preferable to obtain more robust data. We believe that a multicenter study would have been a major challenge. Apart from organizational and planning issues, uncertainties and discrepancies of the level of competence of the instructors may be present. Reliable and valid scoring data would then be dependent on a homogenous, well-experienced, and well-educated teaching staff across the country.

In conclusion, the proposed score sheet seems to have a better potential than the current score sheet as a platform for more accurate scores, avoiding ceiling effects and offering better feedback, and as an instrument for learning and retention of acquired technical surgical skills during Day 1 and Day 2 in the Basic Surgical Skills course. It is suggested that the proposed score sheet replaces the current score sheet in the curriculum for this 3-day long course.
